# Are cardiovascular comorbidities always associated with a worse prognosis in patients with lung cancer?

**DOI:** 10.3389/fcvm.2022.984951

**Published:** 2022-09-23

**Authors:** Sabina Mędrek, Sebastian Szmit

**Affiliations:** ^1^Department of Cardiology, Subcarpathian Oncological Center, Brzozów, Poland; ^2^Department of Pulmonary Circulation, Thromboembolic Diseases and Cardiology, Centre of Postgraduate Medical Education, European Health Centre, Otwock, Poland

**Keywords:** lung cancer, cardio-oncology, survival, prognosis, heart failure, thromboembolism

## Abstract

Many factors contribute to mortality in lung cancer, including the presence of concomitant cardiovascular disease. In the treatment of early stage of lung cancer, the presence of comorbidities and occurence of cardiotoxicity may be prognostic. The effect of cardiotoxicity of radiotherapy and chemoradiotherapy on overall survival has been documented. Acute arterial and venous thromboembolic events seem to correlate with the degree of the histological malignancy, its clinical advancement, and even with optimal cardiac treatment, they may influence the survival time. In the case of high-grade and advanced lung cancer stage especially in an unresectable stadium, the prognosis depends primarily on the factors related to the histopathological and molecular diagnosis. Electrocardiographic and echocardiographic abnormalities may be prognostic factors, as they seem to correlate with the patient's performance status as well as tumor localization and size.

## Introduction

Lung cancer is one of the most common malignancies worldwide and also the most common cause of cancer-related deaths in both men and women. The 5-year survival rates for all stages of lung cancer do not exceed 15–20% (1). There are multiple factors that influence mortality, including the presence of comorbidities. Depending on their duration, these conditions can be classified as chronic and acute.

The Charlson Comorbidity Index (CCI) is the most commonly used tool to assess the likelihood of death within a year for a patient with comorbidities, a score of ≥3 is associated with an 80% increase in the risk of death within a year (Table 1) (2). The CCI is important prognostic scale in oncology because it is independent of cancer stage and performance status. Other scale evaluating comorbidities is the Simplified Comorbidity Score (SCS) confirmed in lung cancer as independent determinant of a poor outcome (Table 1) (3). This validation study revealed the strong statistical concordance between CCI and SCS, by univariate analysis of large group of non-small-cell lung cancer (NSCLC) patients with long term follow up, CCI ≥ 3 and SCS > 9 were considered as important for outcome (*p* = 0.06 and *p* < 0.01 respectively) with final suggestion of higher prognostic value of SCS. However both CCI and SCS were not predictable for survival, radiological response and toxicity during first-line chemotherapy due to advances lung cancer (4). Similarly in patients with advanced and unrespectable NSCLC treated with radical sequential chemoradiotherapy CCI >4 or SCS >8 were not predictors of survival (5).

**Table 1:** The comparison of the main mentioned indexes used for prognosis in lung cancer (2, 3).

**Charlson comorbidity index (CCI)**		**Simplified comorbidity score (SCS)**	
Myocardial infarction	1	Tobacco consumption	7
Congestive heart failure	1	Diabetes mellitus	5
Peripheral vascular disease	1	Renal insufficiency	4
Cerebrovascular disease	1	Respiratory comorbidity	1
Dementia	1	Cardiovascular comorbidity	1
Chronic pulmonary disease	1	Neoplastic comorbidity	1
Connective tissue disease	1	Alcoholism	1
Ulcer disease	1		
Mild liver disease	1		
Diabetes	1		
Hemiplegia	2		
Moderate or severe renal disease	2		
Diabetes with end organ damage	2		
Any tumor without metastasis	2		
Leukemia	2		
Lymphoma	2		
Moderate or severe liver disease	3		
Metastatic solid tumor	6		
AIDS	6		

In patients with NSCLC, cardiovascular (CV) comorbidities, including coronary artery disease, hypertension, arrhythmias, and peripheral arteriosclerosis, increased the risk of death by 30% compared to patients without these conditions (6). However, some studies have shown no direct effect of concomitant diseases on overall survival (OS) in lung cancer in advanced inoperable stage (7). Retrospective cohort studies have shown that cancer-related mortality rates for biologically aggressive malignancies exceed those for comorbidities (8, 9).

Batra et al. showed that patients with lung cancer and CV diseases were less likely to receive oncological treatment, whether chemotherapy, targeted therapy or radiotherapy (10). It has also been shown that prior CV disease increases the risk of cancer-unrelated death (HR = 1.48; *p* < 0.0001) and does not contribute to cancer-related mortality. Disqualification from cancer treatment or use of less intensive and consequently less effective cancer treatment due to comorbidities, which may influence on overall survival, also seem an important problem.

The aim of this study was to review literature on the impact of cardiovascular comorbidities on the prognosis in patients with lung cancer with special focus on the more common NSCLC.

## Cardiac arrhythmias

The mechanism underlying cardiac arrhythmias and conduction disturbances in cancer patients consists of several elements: patient-related factors (comorbidities, age, genetic predisposition), tumor-related factors (invasion, autonomic system excitation, inflammation), cancer treatment-related factors (electrolyte abnormalities due to gastrointestinal toxicity, cardiac structural or electrical remodeling induced by chemotherapy, targeted therapy, immunotherapy, radiotherapy, supportive medications) (11).

Atrial fibrillation (AF) is the most common arrhythmia, affecting 2–4% of the general population, whose prevalence increases with age (up to 36% at the age of 85 years) (12). Cancer patients have many additional reasons to experience AF (13). The OPERA study (Oulu Project Elucidating Risk of Atherosclerosis) showed that cancer disease may be understood an independent risk factor of AF development, because authors recognized AF in 19% with cancer in comparison to only 9% of subject without cancer (*p* < 0.001, HR = 2.47; 95%CI: 1.57–3.88) (14). The new-onset AF may be associated with increased relative risk of a diagnosis of cancer disease of the lung, kidney, colon, ovary as well as non-Hodgkin's lymphoma: seven times increased risk for metastatic and 3.5 times for localized cancer disease (15). The time relationship of 90 days between the recognition of AF and cancer is strongly interesting, because in this period the diagnosis of cancer can predict a 3.4 fold increased risk of new AF, however AF occurrence is related to a 1.85 times higher probability of coexisting cancer (16).

The large population-based study proved that not all types of cancer are associated with AF, but certainly hematological and intrathoracic malignancies are associated with AF (17). This study revealed the risk of AF development is more than doubled in patients with esophageal (HR = 2.69) and lung cancer (HR = 2.39), interestingly, lung cancer showed the strongest association with AF in patients aged > 50 years. Among women with new-onset AF the significant age-adjusted risk was observed for colon (HR = 2.36; *p* < 0.001), breast (HR = 1.35; *p* = 0.04) and lung cancer (HR = 1.69; *p* = 0.04) (18).

Particularly high AF percentages are observed among lung cancer patients. One of the Nationwide population-based study showed the highest incidence of AF as number per person years in lung cancer: 58.7/1000 in men and 35.3/1000 in women (19). The large analysis of cardiovascular admissions to hospital in US revealed AF as the main cause of hospitalization in lung cancer patients and significantly increased mortality was noted in lung cancer and AF (aOR = 4.69) (20).

However, the mechanisms underlying the high incidence of AF in cancer patients are not fully elucidated. In addition to classical risk factors present in the general population (hypertension, diabetes, heart failure, coronary artery disease, obesity, etc.), other factors, i.e., water-electrolyte disturbances, hypoxia, sympathetic over activity due to pain and emotional stress, have also been considered (21, 22). It can be additionally assumed that chronic pulmonary obstructive disease, often coexisting with lung cancer, is also a risk factor for AF, especially if there are episodes of infectious exacerbations and when the size of the left atrium is increased (23). A similar cause can be seen in AF with concomitant pulmonary hypertension associated with hypoxia in lung cancer (24). Compression or infiltration of the tumor mass or metastases to the heart may be another possible cause. AF can also be a complication of systemic treatment, radiotherapy or thoracic surgery (it occurs in 10–20% of patients 2–3 days after surgery) (25).

The prevalence of new onset AF (i.e., first occurrence after the cancer diagnosis) is associated with a higher tumor grade and thus a worse prognosis and higher cardiovascular mortality (26). Poor prognosis has been demonstrated in patients undergoing thoracic surgery for lung cancer who developed AF: increased hospital mortality (6.7 vs. 1.0%, *p* = 0.024) and higher long-term mortality (HR = 3.75) (27). A significant negative prognostic value of AF (HR = 2.39 for mortality, *p* = 0.02) in lung cancer patients qualified for systemic cancer treatment has also been demonstrated (7).

Ventricular arrhythmia is another common type of rhythm abnormalities with possible impact on prognosis in the general population. Anker et al. assessed cancer patients free of cardiovascular diseases in comparison to healthy controls using 24-h ECG (28). They showed an increased frequency of non-sustained ventricular tachycardia (nsVT) observed in 6% of NSCLC patients and associated with negative impact on survival (HR = 2.68; *p* = 0.005). Prognostic value of nsVT was significant independently of type of cancer. Although in the same study single premature ventricular contractions (PVCs) were observed in 42% of NSCLC patients (in 21% in number of 50 or more per 24 h) this arrhythmia did not affect the prognosis for NSCLC patients, but surprisingly it had an effect on the survival of patients with pancreatic and colorectal cancer. As one of possible explanation may be a fact that NSCLC patients receive beta-blockers more frequent which could inhibit PVCs. In the larger study where 24-h ECG recordings from the period of 6 years (2012–2018) were reviewed, the highest frequency of nsVT (33%) was observed in lung cancer patients without cardiac dysfunction (29). Moreover 52% of lung cancer patients had at least 20 PVCs during monitoring. The analysis in whole group of cancer patients revealed the arrhythmias nsVT ≥ 4 beats or PVCs ≥ 20/day were independently associated with higher risk of all-cause mortality (HR = 1.81, *p* = 0.016 and HR = 1.6, *p* = 0.0088, respectively in multivariate adjusted analysis).

Elevated resting heart rate alone may be an independent risk factor for death in stable cardiovascular disease in general cardiology (30). In selected cancer diseases irrespective of hemoglobin levels or tumor grade, a similar relationship has been observed (31). Hemu et al. showed that sinus tachycardia (heart rate ≥100 /min.) occurring during cancer treatment is associated with an increase in cardiovascular events and mortality over a 10-year period (32). The prospective study in lung cancer demonstrated the prognostic significance of heart rate, regardless of whether it was sinus rhythm or atrial fibrillation, heart rate > 90/min predicted a higher risk of mortality (HR = 1.67; *p* = 0.03) (7). The tumor growth effect can be considered a potential explanation, as shorter survival was also observed in patients with right ventricular systolic pressure (RVSP) higher than 39 mmHg (HR = 2.01; *p* = 0.0045).

On the other hand, asymptomatic sinus bradycardia, defined as heart rate < 50/min, is an adverse effect of ALK inhibitor treatment (such as crizotinib) and may positively correlate with clinical response to treatment (33, 34). However it should be remembered that this slowing of the heart rate is an effect of the cancer drug activity and therefore appears to correlate with its efficacy.

## Arterial hypertension

Hypertension (HT) is one of the major single risk factors for cardiovascular (CV) diseases and increased CV mortality (35). A worldwide survey among 1.5 million adults performed in May 2019 showed that 32% of the population had never had their blood pressure measured, 34% had HT of whom 23.3% had untreated or sub-optimally treated HT (36). It is also the most common comorbidity among cancer patients, regardless of the type of malignancy identified in 38% of patients (37). Prospective multicenter study documented HT in 43% of NSCLC patients (38). Similarly Polish study based on one center experience identified HT in 42.3% of metastatic NSCLC EGFR positive patients (39).

There are no papers clearly demonstrating the negative impact of pre-existing HT as a single prognostic factor in lung cancer. Moreover, there are no evidences that lung cancer (except for neuroendocrine type) may lead to HT development.

Lung cancer patients usually experience increase in blood pressure related to cancer therapy. Cisplatin is the most commonly used alkylating agent in various treatment regimens for both NSCLC and small cell lung cancer (SCLC). It increases blood pressure at varying rates, depending on the observation of patients with testicular cancer: 39% in 10-year follow-up (40) or 53% in 11-year follow-up (41). The main mechanisms leading to increased blood pressure include endothelial cell dysfunction or damage, excessive platelet aggregation and reduced nitric oxide availability (42). Anti-VEGF agents, such as bevacizumab and ramucirumab, are other drugs used in the treatment of these tumors that also may contribute to the increase in blood pressure. Yan et al. showed that HT in the treatment with bevacizumab-based regimens for metastatic NSCLC was associated with higher response rates (43). This is a next clear confirmation that HT as effect of cancer drug activity correlate positively with outcome. Supportive therapies, such as steroids, non-steroidal anti-inflammatory drugs or erythropoietin, are also implicated in hypertension (44). Reduction of angiotensin-converting enzyme (ACE) activity in tumor tissue correlating with poor prognosis and tumor metastasis is yet another problem. One retrospective paper has shown a positive effect of RAAS blockers on survival, whereas none has shown its negative effect (45).

### Coronary artery disease

Coronary artery disease (CAD) is a condition associated with the formation of atherosclerotic plaques in the coronary arteries, which runs with periods of clinical stability (chronic coronary syndrome) and destabilization (acute coronary syndrome). According to population-based studies, the CAD prevalence increases with age and is 10–12% in women aged 65–84 years, and 12–14% in similarly aged men (46). The prevalence in lung cancer patients seems to be higher because ranges from 10.3% (10) to 33.7% (47), depending on the source. Every third patient (33%) from those with cardiovascular disease had previous myocardial infarction (10).

The high prevalence of CAD and lung cancer is due to common aetiological factors: cigarette smoking (48), advanced age, obesity (49) and the same pathomechanism associated with oxidative stress and chronic inflammation (50, 51). During lung cancer screening by a low-dose CT scan, coronary micro-calcifications indicative of atherosclerotic lesions have been additionally found as often coexisting clinical problem (52, 53). Sun et al. demonstrated a relationship between the severity of CAD (degree of coronary stenosis) and lung cancer, which may broaden the diagnostic scope for this malignancy in the future (54).

The coexistence of CAD can worsen the prognosis of patients undergoing surgery for stage I and II NSCLC (55, 56). On the other hand, other studies have shown no significant effect of CV disease on mortality during primary surgery (57, 58). Assuming that CAD is the most common cause of CV diseases, the conflicting data may reflect the thoracic perioperative risk and prognosis may depend on the severity of CAD and control of ischemic symptoms through effective cardiac treatment.

Acute coronary syndromes (ACS) are another problem. Approximately 15% of patients treated for ACS have a coexisting cancer (59). Non-ST-segment elevation myocardial infarction (NSTEMI) accounted for the majority of ACS in cancer patients, as in the general population (60). The clinical picture of ACS in cancer patients is untypical, with only 33% of patients experiencing chest pain, 44% reporting dyspnoea and 23% developing hypotension (61). ACS can also be triggered by anti-cancer treatment. Cisplatin, gemcitabine and bevacizumab are highly thrombogenic (62). Treatment guidelines for cancer patients are missing, and it is believed that this group should be treated like other patients.

Many studies show a worse prognosis for patients with ACS and cancer (63–65). In particular, lung cancer patients are at risk of arterial thromboembolism like myocardial infarction and in this way the risk of mortality is increased three times (66). Lung cancer is one of the four most common types of cancer disease with highest frequency of myocardial infarction, only 21.0% of those patients were treated by coronary intervention, lung cancer was associated with the highest in-hospital mortality, major adverse cardiovascular and cerebrovascular complications (67). The large “real world” data on prognosis after STEMI presented lung cancer as one of the strongest independent determinants of all-cause mortality (HR = 2.04), next advanced peripheral artery disease (HR = 1.78), metastasis (HR = 1.72), previous stroke (HR = 1.44) (68).

### Heart failure

Heart failure (HF) affects 1–2% of adults in the general population, and its prevalence increases with age (69). There are 23 million people worldwide with HF (70) and many of them may experience of lung cancer.

Current treatment of HF improves patients' survival, which means that more of these patients will have cancer. In an analysis performed by the Women's Health Initiative, in postmenopausal women HF was shown to be associated with an increased incidence of obesity-related cancers (HR = 1.24), and even more with the risk of developing lung cancer (HR = 1.58) (71). The prevalence of HF in patients with lung cancer is estimated between 7.6% (72) and 17.5% (47), depending on the source.

HF patients were less likely to undergo surgery and chemotherapy than patients without HF. HF is significantly correlated with increased perioperative mortality in lung cancer (OR = 6.0) (73). More and more aggressive anticancer treatment will increase the number of patients with newly diagnosed HF (74). Both old and newer drugs recommended in lung cancer seem to predispose to HF development through different patomechanism (75, 76).

Patients with known lung cancer hospitalized for HF have a higher mortality rate (5.9%) compared to those cancer-free (3.3%) (77). In the same study, as a very optimistic observation, it should be considered that over the years from 2003 to 2014, mortality among patients hospitalized due to HF decreased, but very importantly, the decrease in mortality was the highest among patients with accompanying lung cancer (8.1 to 4.6%; *p* < 0.001).

### Valvular heart disease

Valvular heart disease (VHD) may occur in cancer patients for several reasons: due to pre-existing valvular defects and as a complication of anti-cancer treatment: after radiotherapy, due to infective endocarditis in the course of chemotherapy-induced severe infection and secondary to cardiac dysfunction (78, 79).

Degenerative aortic valve stenosis is the most common primary valvular heart defect in the general population (80). The majority of patients with active cancer are disqualified from classical cardiac surgery (surgical aortic valve replacement, SAVR) due to the high risk of perioperative complications such as bleeding, arrhythmias, infections or coagulation disorders (81). Transcatheter aortic valve replacement (TAVR) seems to be an alternative solution. According to current European recommendations, the procedure should be performed in patients with expected survival of at least 1 year (82), but only a small percentage of patients with advanced lung cancer meets such criteria. In a study by Landes et al. compared the survival of patients with and without cancer who underwent TAVR, lung cancer patients constituted a small group - only 11% (83). The authors showed a worse prognosis in oncology patients, with tumor stage being the strongest predictor of late mortality. Similar findings were also published in several other papers (84–86). However it is worth to consider concomitant cardiac surgery as treatment option for valvular defects in patients with early stage lung cancer scheduled for thoracic surgery (87).

The prevalence of radiotherapy-induced VHD is described as frequent, affecting approximately 10% of treated patients (88, 89), but it occurs late (median time to diagnosis is 22 years) (90). The short expected survival time of lung cancer patients does not allow for the manifestation of late cardiac toxicity, which is a typical complication of radiation therapy.

Cancer patients have a higher risk of developing infective endocarditis (IE) due to immunosuppression (e.g., secondary to chemotherapy) or the presence of a central line or a vascular port (91). In most studies, *Staphylococcus aureus* was the predominant aetiological agent, with one native valve (aortic or mitral, less frequently tricuspid) most frequently involved (92). A higher mortality in the course of IE was also demonstrated in all cancer patients in comparison to the control group (also associated with tumor progression). Cardiac surgery was performed in approximately 50% of patients (93, 94).

## Venous thromboembolism

Venous thromboembolism (VTE) as deep vein thrombosis (DVT) and pulmonary embolism (PE) is the common clinical worldwide problem in general population, because last data showed the annual incidence rate of VTE in Europe ranged from 104 to 183 per 100000 person-years (95). Prevalence of VTE varies greatly from region to region but generally fluctuates 39–115/100000 for PE, and 53–162/100000 for DVT (96). Acute PE remains the third most common cause of acute cardiovascular syndromes, its incidence increases (97, 98).

Major surgery (OR = 18.95) and active cancer (OR = 14.64) belong to the strongest independent risk factors for DVT or PE (99). Lung cancer is the sixth most frequent reason of PE among malignancies (100). Lung cancer, especially adenocarcinoma, predisposes to PE more than other malignancies, especially within 3 months of the diagnosis (101). It has also been shown that lung cancer patients are six times more likely to develop PE than those cancer-free in the 12 months preceding the diagnosis (102). Risk of PE diagnosis correlates with a moment of cancer occurrence: for NSCLC: HR = 9.7 during 6 months prior to cancer diagnosis, HR = 20.0 during 6 months after cancer diagnosis and HR = 17.4 during 12 months after cancer diagnosis and for SCLC: HR = 6.9 and HR = 14.8 and HR = 16.1 respectively (103).

PE most often accompanies advanced-stage lung cancer (stages III to IV) (104). The Vienna Cancer and Thrombosis Study by multivariable Cox proportional hazards analysis confirmed that lung is one of the high risk tumor site associated with VTE (HR = 4.3; *p* < 0.001) together with high tumor grade, tumor histology (adenocarcinoma) and elevated D-dimer level (105). PE is diagnosed in a high percentage of cancer patients incidentally as unprovoked PE or asymptomatic PE (the increased risk of incidental PE in cancer was calculated as OR = 1.80) (106). PE can be recognized during diagnostic imaging for staging or evaluation of response to cancer treatment. In lung cancer such correlation with asymptomatic/incidental PE ranges from 29.4 to 63% of patients (107). Colorectal cancer and lung cancer appears to be two cancer diseases with the most frequent incidental VTE (108).

Lung cancer treatment can induce new episodes of VTE (107). PE developed during lung cancer treatment rather does not affect survival (*p* = 0.206) (101). Subsequent cancer remission resulted from cancer therapy and control of cancer-related coagulation state seem to reduce the occurrence of VTE important for prognosis (109).

Nichols et al. showed in their post-mortem studies in lung cancer patients that PE was the direct cause of death in 10% of cases, however from a pathophysiologic perspective, PE may be an additional contributing cause of death in many other cases (110). PE significantly worsens the prognosis in lung cancer (*p* < 0.0005) and as a possible explanation authors discussed more advanced stage of cancer disease (III or IV) and more frequent used only supportive care without anticancer therapy (111). In the prospective cohort study in older patients (age ≥ 65 years) with lung cancer it was documented significantly shorter survival in subgroup with PE (4.3 vs. 9.2 months, *p* = 0.0015), there were significant differences in PE-related mortality (15.1 vs. 0%) but insignificant differences in tumor-related mortality (75.5 vs. 66.0%) (112). It should be highlighted that PE is associated with shorter survival when is recognized synchronous with lung cancer (113).

No difference in mortality between symptomatic vs. asymptomatic PE in lung cancer was documented, with both forms worsening the prognosis due to haemorrhagic complications and VTE recurrences (there were similar patients' age and frequency of metastatic disease) (114). There are data that even 55% of lung cancer with unsuspected PE did not receive anticoagulation therapy which leaded to premature death (HR = 4.1) (115).

### Comorbidity or multi-morbidity

The outcome and quality of life in lung cancer can be determine not only by coexisting cardiovascular diseases, the importance of other age-, obesity- and tobacco-related diseases should be taken into account. Number of comorbidities in lung cancer is so high that authors from Spain proposed the term multi-morbidity when at least two chronic diseases coexist with lung cancer and documented the highest mortality in patients with multi-morbidity (*p* = 0.002) in comparison to patients with one or no comorbidity (40% higher mortality) (116). It is worth emphasizing that the prevalence of multi-morbidity correlated with older patients' age and history of smoking.

Apart from cardiovascular diseases, chronic obstructive pulmonary disease is most often associated with lung cancer (117). Among other serious co-morbidities another cancer (10–20%) and diabetes mellitus (5–25%) seem to be essential for prognosis (118). Generally, mortality in lung cancer was defined as 1.1–1.5 times higher for patients with comorbidity (119). In lung cancer 19 comorbidities were found as independent predictors of survival (72). The Nebraska Hospital Discharge Data showed survival in lung cancer may negatively depend on congestive HF, diabetes, liver disease, dementia, renal disease, cerebrovascular disease, the greatest difference in survival in patients with and without comorbidities was seen at low grades: HR = 1.316 for localized, HR = 1.228 for regional and HR = 1.075 for metastatic lung cancer (120). Due to frequent follow-up, patients with comorbidities were more likely to be diagnosed at an early stages of each cancer disease.

## Conclusion

**Figure 1:**
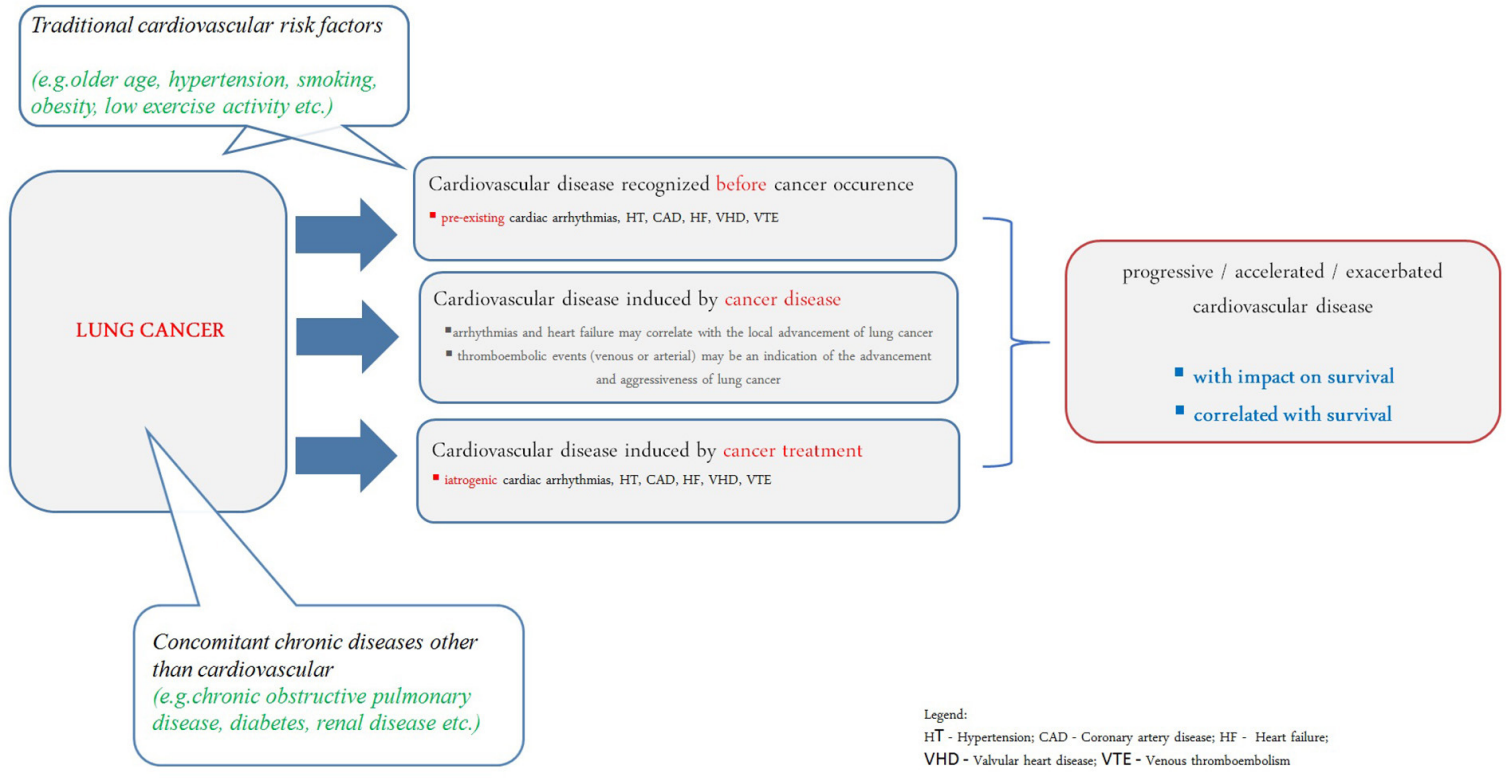
Understanding of cardiovascular disease in prognosis of lung cancer patients. HT, hypertension; CAD, coronary artery disease; HF, heart failure; VHD, valvular heart disease; VTE, venous thromboembolism.

Acute cardiac conditions, such as pulmonary embolism or myocardial infarction, clearly worsen the prognosis in lung cancer. Lung cancer belongs to such malignancies where the risk of venous and arterial thromboembolic complications correlates with tumor advancement (Figure 1) (105, 121). Often an arterial or venous thromboembolic event occurs at the onset of the neoplastic disease (122, 123).

Systemic treatment and radiotherapy in lung cancer may cause cardiovascular complications (124, 125). It has been shown that in patients over 65 years of age undergoing chemotherapy, the risk of developing CAD or HF was increased; cardiac disorders were also more common in patients undergoing radiotherapy, especially if the left lung was irradiated. The greatest risk of cardiotoxicity was found in patients undergoing chemo-radiotherapy (126). ARIC Study revealed that lung cancer survivors have higher risk of cardiovascular disease development (especially HF) even they do not have traditional cardiovascular risk factors (Figure 1) (127).

Lung cancer patients have the highest prevalence of cardiovascular comorbidities compared to other cancers (128). At least one concomitant cardiovascular disease was present in 67.2% of patients with NSCLC (129). The hypothesis that the effect of chronic cardiac comorbidities on mortality is dominant in early stages of lung cancer seems most plausible. Data on 95 167 NSCLC patients showed that cardiovascular disease can increase mortality when the cancer stage is in the range I-III B, while it is not important for survival in stage IV (10). Worse prognosis was associated with concomitant heart failure, myocardial infarction, and arrhythmias diagnosed during follow-up, although the risk still varied depending on the stage of the disease and the treatment method. For stage I-IIIB disease, concomitant cardiovascular diseases increased the risk of mortality by as much as 2.59 (*p* < 0.001) for chemotherapy and by 2.20 (*p* < 0.001) for chemotherapy and radiotherapy.

The impact of cardiovascular comorbidities on prognosis is limited in advanced stages of lung cancer. Cardiac arrhythmias (especially atrial fibrillation) and echocardiographic changes suggesting the development of pulmonary hypertension (right ventricular systolic pressure increase) and dysfunction of the right ventricle rather result from the advancement of neoplastic disease, correlate with decreased performance status and predict shorter overall survival (Figure 1) (7).

## Author contributions

All authors have design and conception, writing of manuscript, editing and reviewing the manuscript and final approval of the version to be published.

## Conflict of interest

SS declares speaker fee or advisory board fee or travel and meeting support: Angelini, Amgen, Astra-Zeneca, Bayer, BMS, Gilead, Pfizer, TEVA (all outside of this study). The remaining author declare that the research was conducted in the absence of any commercial or financial relationships that could be construed as a potential conflict of interest.

## Publisher's note

All claims expressed in this article are solely those of the authors and do not necessarily represent those of their affiliated organizations, or those of the publisher, the editors and the reviewers. Any product that may be evaluated in this article, or claim that may be made by its manufacturer, is not guaranteed or endorsed by the publisher.

## References

[1] Siegel RL Miller KD Fuchs HE Jemal A. Cancer Statistics, 2021. CA Cancer J Clin. (2021) 71:7–33. 10.3322/caac.21654 33433946

[2] Charlson ME Pompei P Ales KL MacKenzie CR. A new method of classifying prognostic comorbidity in longitudinal studies: development and validation. J Chronic Dis. (1987) 40:373–83. 10.1016/0021-9681(87)90171-8 3558716

[3] Colinet B Jacot W Bertrand D Lacombe S Bozonnat MC Daurès JP. oncoLR health network. A new simplified comorbidity score as a prognostic factor in non-small-cell lung cancer patients: description and comparison with the Charlson's index. Br J Cancer. (2005) 93:1098–105. 10.1038/sj.bjc.6602836 16234816 PMC2361505

[4] Singh N Singh PS Aggarwal AN Behera D. Comorbidity assessment using charlson comorbidity index and simplified comorbidity score and its association with clinical outcomes during first-line chemotherapy for lung cancer. Clin Lung Cancer. (2016) 17:205–213.e1. 10.1016/j.cllc.2015.10.002 26589440

[5] Zaborowska-Szmit M Olszyna-Serementa M Kowalski DM Szmit S Krzakowski M. Elderly patients with locally advanced and unresectable non-small-cell lung cancer may benefit from sequential chemoradiotherapy. Cancers. (2021) 13:4534. 10.3390/cancers13184534 34572760 PMC8466795

[6] Iachina M Jakobsen E Møller H Lüchtenborg M Mellemgaard A Krasnik M. The effect of different comorbidities on survival of non-small cells lung cancer patients. Lung. (2015) 193:291–7. 10.1007/s00408-014-9675-5 25516286

[7] Medrek S Szmit S. Baseline electrocardiographic and echocardiographic assessment may help predict survival in lung cancer patients-a prospective cardio-oncology study. Cancers. (2022) 14:2010. 10.3390/cancers14082010 35454916 PMC9032028

[8] Kendal WS. Dying with cancer: the influence of age, comorbidity, and cancer site. Cancer. (2008) 112:1354–62. 10.1002/cncr.23315 18286532

[9] Zaorsky NG Churilla TM Egleston BL Fisher SG Ridge JA Horwitz EM. Causes of death among cancer patients. Ann Oncol. (2017) 28:400–7. 10.1093/annonc/mdw604 27831506 PMC5834100

[10] Batra A Sheka D Kong S Cheung WY. Impact of pre-existing cardiovascular disease on treatment patterns and survival outcomes in patients with lung cancer. BMC Cancer. (2020) 20:1004. 10.1186/s12885-020-07487-9 33059611 PMC7559447

[11] Farmakis D Filippatos G. Arrhythmias in cancer: rhythm is gonna get you! Eur J Heart Fail. (2021) 23:154–6. 10.1002/ejhf.2079 33340386

[12] Benjamin EJ Muntner P Alonso A Bittencourt MS Callaway CW Carson AP. Heart disease and stroke statistics-2019 update: a report from the American Heart Association. Circulation. (2019) 139:e56–e528. 10.1161/CIR.0000000000000659 30700139

[13] Chu G Versteeg HH Verschoor AJ Trines SA Hemels MEW Ay C. Atrial fibrillation and cancer - An unexplored field in cardiovascular oncology. Blood Rev. (2019) 35:59–67. 10.1016/j.blre.2019.03.005 30928168

[14] Kattelus H Kesäniemi YA Huikuri H Ukkola O. Cancer increases the risk of atrial fibrillation during long-term follow-up (OPERA study). PLoS ONE. (2018) 13:e0205454. 10.1371/journal.pone.0205454 30289944 PMC6173458

[15] Ostenfeld EB Erichsen R Pedersen L Farkas DK Weiss NS Sørensen HT. Atrial fibrillation as a marker of occult cancer. PLoS ONE. (2014) 9:e102861. 10.1371/journal.pone.0102861 25119880 PMC4138009

[16] Saliba W Rennert HS Gronich N Gruber SB Rennert G. Association of atrial fibrillation and cancer: Analysis from two large population-based case-control studies. PLoS ONE. (2018) 13:e0190324. 10.1371/journal.pone.0190324 29324747 PMC5764256

[17] Yun JP Choi EK Han KD Park SH Jung JH Park SH. Risk of atrial fibrillation according to cancer type: a nationwide population-based study. JACC CardioOncol. (2021) 3:221–32. 10.1016/j.jaccao.2021.03.006 34396327 PMC8352078

[18] Conen D Wong JA Sandhu RK Cook NR Lee IM Buring JE. Risk of malignant cancer among women with new-onset atrial fibrillation. JAMA Cardiol. (2016) 1:389–96. 10.1001/jamacardio.2016.0280 27438314 PMC4957657

[19] Jakobsen CB Lamberts M Carlson N Lock-Hansen M Torp-Pedersen C Gislason GH. Incidence of atrial fibrillation in different major cancer subtypes: a Nationwide population-based 12 year follow up study. BMC Cancer. (2019) 19:1105. 10.1186/s12885-019-6314-9 31726997 PMC6854796

[20] Matetic A Mohamed M Miller RJH Kolman L Lopez-Mattei J Cheung WY. Impact of cancer diagnosis on causes and outcomes of 59 million US patients with cardiovascular admissions. Int J Cardiol. (2021) 341:76–83. 10.1016/j.ijcard.2021.07.054 34333019

[21] Farmakis D Parissis J Filippatos G. Insights into onco-cardiology: atrial fibrillation in cancer. J Am Coll Cardiol. (2014) 63:945–53. 10.1016/j.jacc.2013.11.026 24361314

[22] Chen PS Chen LS Fishbein MC Lin SF Nattel S. Role of the autonomic nervous system in atrial fibrillation: pathophysiology and therapy. Circ Res. (2014) 114:1500–15. 10.1161/CIRCRESAHA.114.303772 24763467 PMC4043633

[23] Grymonprez M Vakaet V Kavousi M Stricker BH Ikram MA Heeringa J. Chronic obstructive pulmonary disease and the development of atrial fibrillation. Int J Cardiol. (2019) 276:118–24. 10.1016/j.ijcard.2018.09.056 30268382

[24] Wanamaker B Cascino T McLaughlin V Oral H Latchamsetty R Siontis KC. Atrial Arrhythmias in Pulmonary Hypertension: Pathogenesis, Prognosis and Management. Arrhythm Electrophysiol Rev. (2018) 7:43–8. 10.15420/aer.2018.3.2 29636972 PMC5889803

[25] Bandyopadhyay D Ball S Hajra A Chakraborty S Dey AK Ghosh RK. Impact of atrial fibrillation in patients with lung cancer: Insights from National Inpatient Sample. Int J Cardiol Heart Vasc. (2019) 22:216–7. 10.1016/j.ijcha.2019.02.012 30963100 PMC6437280

[26] Yang X Li X Yuan M Tian C Yang Y Wang X. Anticancer therapy-induced atrial fibrillation: electrophysiology and related mechanisms. Front Pharmacol. (2018) 9:1058. 10.3389/fphar.2018.01058 30386232 PMC6198283

[27] Imperatori A Mariscalco G Riganti G Rotolo N Conti V Dominioni L. Atrial fibrillation after pulmonary lobectomy for lung cancer affects long-term survival in a prospective single-center study. J Cardiothorac Surg. (2012) 7:4. 10.1186/1749-8090-7-4 22233837 PMC3287133

[28] Anker MS von Haehling S Coats AJS Riess H Eucker J Porthun J. Ventricular tachycardia, premature ventricular contractions, and mortality in unselected patients with lung, colon, or pancreatic cancer: a prospective study. Eur J Heart Fail. (2021) 23:145–53. 10.1002/ejhf.2059 33222388

[29] Albrecht A Porthun J Eucker J Coats AJS von Haehling S Pezzutto A. Spontaneous non-sustained ventricular tachycardia and premature ventricular contractions and their prognostic relevance in patients with cancer in routine care. Cancers. (2021) 13:2303. 10.3390/cancers13102303 34065780 PMC8151948

[30] Lonn EM Rambihar S Gao P Custodis FF Sliwa K Teo KK. Heart rate is associated with increased risk of major cardiovascular events, cardiovascular and all-cause death in patients with stable chronic cardiovascular disease: an analysis of ONTARGET/TRANSCEND. Clin Res Cardiol. (2014) 103:149–59. 10.1007/s00392-013-0644-4 24356937

[31] Anker MS Ebner N Hildebrandt B Springer J Sinn M Riess H. Resting heart rate is an independent predictor of death in patients with colorectal, pancreatic, and non-small cell lung cancer: results of a prospective cardiovascular long-term study. Eur J Heart Fail. (2016) 18:1524–34. 10.1002/ejhf.670 27910284

[32] Hemu M Chiang CJ Bhatt PK Ahmed A Hein KZ Mourad T. Associations between sinus tachycardia and adverse cardiovascular outcomes and mortality in cancer patients. J Thorac Dis. (2021) 13:4845–52. 10.21037/jtd-21-779 34527323 PMC8411161

[33] Ou SH Tong WP Azada M Siwak-Tapp C Dy J Stiber JA. Heart rate decrease during crizotinib treatment and potential correlation to clinical response. Cancer. (2013) 119:1969–75. 10.1002/cncr.28040 23505007

[34] Ou SH Azada M Dy J Stiber JA. Asymptomatic profound sinus bradycardia (heart rate ≤ 45) in non-small cell lung cancer patients treated with crizotinib. J Thorac Oncol. (2011) 6:2135–7. 10.1097/JTO.0b013e3182307e06 22088989

[35] Roth GA Mensah GA Johnson CO Addolorato G Ammirati E Baddour LM. Global burden of cardiovascular diseases and risk factors, 1990-2019: update from the GBD 2019 study. J Am Coll Cardiol. (2020) 76:2982–3021. 33309175 10.1016/j.jacc.2020.11.010 PMC7755038

[36] Beaney T Schutte AE Stergiou GS Borghi C Burger D Charchar F. May measurement month 2019: the global blood pressure screening campaign of the international society of hypertension. Hypertension. (2020) 76:333–41. 10.1161/HYPERTENSIONAHA.120.14874 32419505

[37] Piccirillo JF Tierney RM Costas I Grove L Spitznagel EL. Prognostic importance of comorbidity in a hospital-based cancer registry. JAMA. (2004) 291:2441–7. 10.1001/jama.291.20.2441 15161894

[38] Herrero Rivera D Nieto-Guerrero Gómez JM Cacicedo Fernández de Bobadilla J Delgado D Rivin Del Campo E Praena-Fernández JM. Cardiovascular disease and survival in non-small cell lung cancer: a multicenter prospective assessment. Clin Transl Oncol. (2019) 21:1220–30. 10.1007/s12094-019-02047-5 30680608

[39] Zaborowska-Szmit M Kowalski DM Piórek A Krzakowski M Szmit S. A decrease in D-dimer concentration and an occurrence of skin rash as iatrogenic events and complementary predictors of survival in lung cancer patients treated with EGFR tyrosine kinase inhibitors. Pharmacol Rep. (2016) 68:1140–8. 10.1016/j.pharep.2016.07.003 27588390

[40] Meinardi MT Gietema JA van der Graaf WT van Veldhuisen DJ Runne MA Sluiter WJ. Cardiovascular morbidity in long-term survivors of metastatic testicular cancer. J Clin Oncol. (2000) 18:1725–32. 10.1200/JCO.2000.18.8.1725 10764433

[41] Sagstuen H Aass N Fosså SD Dahl O Klepp O Wist EA. Blood pressure and body mass index in long-term survivors of testicular cancer. J Clin Oncol. (2005) 23:4980–90. 10.1200/JCO.2005.06.882 16051950

[42] Soultati A Mountzios G Avgerinou C Papaxoinis G Pectasides D Dimopoulos MA. Endothelial vascular toxicity from chemotherapeutic agents: preclinical evidence and clinical implications. Cancer Treat Rev. (2012) 38:473–83. 10.1016/j.ctrv.2011.09.002 21982720

[43] Yan LZ Dressler EV Adams VR. Association of hypertension and treatment outcomes in advanced stage non-small cell lung cancer patients treated with bevacizumab or non-bevacizumab containing regimens. J Oncol Pharm Pract. (2018) 24:209–17. 10.1177/1078155217690921 29284349

[44] Grossman A Messerli FH Grossman E. Drug induced hypertension–An unappreciated cause of secondary hypertension. Eur J Pharmacol. (2015) 763:15–22. 10.1016/j.ejphar.2015.06.027 26096556

[45] Rachow T Schiffl H Lang SM. Risk of lung cancer and renin-angiotensin blockade: a concise review. J Cancer Res Clin Oncol. (2021) 147:195–204. 10.1007/s00432-020-03445-x 33231730 PMC7684567

[46] Knuuti J Wijns W Saraste A Capodanno D Barbato E Funck-Brentano C. 2019 ESC Guidelines for the diagnosis and management of chronic coronary syndromes. Eur Heart J. (2020) 41:407–77. 10.1093/eurheartj/ehz425 31504439

[47] Kravchenko J Berry M Arbeev K Lyerly HK Yashin A Akushevich I. Cardiovascular comorbidities and survival of lung cancer patients: Medicare data based analysis. Lung Cancer. (2015) 88:85–93. 10.1016/j.lungcan.2015.01.006 25704956 PMC4375130

[48] Alberg AJ Samet JM. Epidemiology of lung cancer. Chest. (2003) 123:21S−49S. 10.1378/chest.123.1_suppl.21S 12527563

[49] Shields M Carroll MD Ogden CL. Adult obesity prevalence in Canada and the United States. NCHS Data Brief. (2011) 56:1–8. 21592419

[50] Barrera G. Oxidative stress and lipid peroxidation products in cancer progression and therapy. ISRN Oncol. (2012) 2012:137289. 10.5402/2012/137289 23119185 PMC3483701

[51] Kamp DW Shacter E Weitzman SA. Chronic inflammation and cancer: the role of the mitochondria. Oncology. (2011) 25:400–10. 21710835

[52] Arcadi T Maffei E Sverzellati N Mantini C Guaricci AI Tedeschi C. Coronary artery calcium score on low-dose computed tomography for lung cancer screening. World J Radiol. (2014) 6:381–7. 10.4329/wjr.v6.i6.381 24976939 PMC4072823

[53] Mendoza DP Kako B Digumarthy SR Shepard JO Little BP. Impact of significant coronary artery calcification reported on low-dose computed tomography lung cancer screening. J Thorac Imaging. (2020) 35:129–35. 10.1097/RTI.0000000000000458 31651689

[54] Sun M Yang Q Li M Jing J Zhou H Chen Y. Association between the severity of coronary artery disease and lung cancer: a pilot cross-sectional study. Arq Bras Cardiol. (2022) 118:478–85. 10.36660/abc.20200478 35262584 PMC8856686

[55] Licker M de Perrot M Höhn L Tschopp JM Robert J Frey JG. Perioperative mortality and major cardio-pulmonary complications after lung surgery for non-small cell carcinoma. Eur J Cardiothorac Surg. (1999) 15:314–9. 10.1016/S1010-7940(99)00006-8 10333029

[56] Ambrogi V Pompeo E Elia S Pistolese GR Mineo TC. The impact of cardiovascular comorbidity on the outcome of surgery for stage I and II non-small-cell lung cancer. Eur J Cardiothorac Surg. (2003) 23:811–7. 10.1016/S1010-7940(03)00093-9 12754038

[57] Mishra PK Pandey R Shackcloth MJ McShane J Grayson AD Carr MH. Cardiac comorbidity is not a risk factor for mortality and morbidity following surgery for primary non-small cell lung cancer. Eur J Cardiothorac Surg. (2009) 35:439–43. 10.1016/j.ejcts.2008.10.029 19081729

[58] Takenaka T Katsura M Shikada Y Tsukamoto S Takeo S. The impact of cardiovascular comorbidities on the outcome of surgery for non-small-cell lung cancer. Interact Cardiovasc Thorac Surg. (2013) 16:270–4. 10.1093/icvts/ivs489 23223675 PMC3568808

[59] Chen HY Saczynski JS McManus DD Lessard D Yarzebski J Lapane KL. The impact of cardiac and noncardiac comorbidities on the short-term outcomes of patients hospitalized with acute myocardial infarction: a population-based perspective. Clin Epidemiol. (2013) 5:439–48. 10.2147/CLEP.S49485 24235847 PMC3825685

[60] Guha A Dey AK Jneid H Addison D. Acute coronary syndromes in cancer patients. Eur Heart J. (2019) 40:1487–90. 10.1093/eurheartj/ehz267 31087050 PMC6821352

[61] Banasiak W Zymliński R Undas A. Optimal management of cancer patients with acute coronary syndrome. Pol Arch Intern Med. (2018) 128:244–53. 10.20452/pamw.4254 29708956

[62] Zamorano JL Lancellotti P Rodriguez Muñoz D Aboyans V Asteggiano R Galderisi M. 2016 ESC Position Paper on cancer treatments and cardiovascular toxicity developed under the auspices of the ESC Committee for Practice Guidelines: The Task Force for cancer treatments and cardiovascular toxicity of the European Society of Cardiology (ESC). Eur Heart J. (2016) 37:2768–801. 10.1093/eurheartj/ehw211 27567406

[63] Landes U Kornowski R Bental T Assali A Vaknin-Assa H Lev E. Long-term outcomes after percutaneous coronary interventions in cancer survivors. Coron Artery Dis. (2017) 28:5–10. 10.1097/MCA.0000000000000429 27622995

[64] Rohrmann S Witassek F Erne P Rickli H Radovanovic D. Treatment of patients with myocardial infarction depends on history of cancer. Eur Heart J Acute Cardiovasc Care. (2018) 7:639–45. 10.1177/2048872617729636 28927294

[65] Potts JE Iliescu CA Lopez Mattei JC Martinez SC Holmvang L Ludman P. Percutaneous coronary intervention in cancer patients: a report of the prevalence and outcomes in the United States. Eur Heart J. (2019) 40:1790–800. 10.1093/eurheartj/ehy769 30500952

[66] Grilz E Königsbrügge O Posch F Schmidinger M Pirker R Lang IM. Frequency, risk factors, and impact on mortality of arterial thromboembolism in patients with cancer. Haematologica. (2018) 103:1549–56. 10.3324/haematol.2018.192419 29794142 PMC6119137

[67] Bharadwaj A Potts J Mohamed MO Parwani P Swamy P Lopez-Mattei JC. Acute myocardial infarction treatments and outcomes in 65 million patients with a current or historical diagnosis of cancer in the USA. Eur Heart J. (2020) 41:2183–93. 10.1093/eurheartj/ehz851 31800032

[68] Lange SA Feld J Kühnemund L Köppe J Makowski L Engelbertz CM. Acute and long-term outcomes of ST-elevation myocardial infarction in cancer patients, a 'real world' analysis with 175,000 patients. Cancers. (2021) 13:6203. 10.3390/cancers13246203 34944823 PMC8699199

[69] Ponikowski P Anker SD AlHabib KF Cowie MR Force TL Hu S. Heart failure: preventing disease and death worldwide. ESC Heart Fail. (2014) 1:4–25. 10.1002/ehf2.12005 28834669

[70] Bui AL Horwich TB Fonarow GC. Epidemiology and risk profile of heart failure. Nat Rev Cardiol. (2011) 8:30–41. 10.1038/nrcardio.2010.165 21060326 PMC3033496

[71] Leedy DJ Reding KW Vasbinder AL Anderson GL Barac A Wactawski-Wende J. The association between heart failure and incident cancer in women: an analysis of the Women's Health Initiative. Eur J Heart Fail. (2021) 23:1712–21. 10.1002/ejhf.2207 33932263 PMC8591611

[72] Tammemagi CM Neslund-Dudas C Simoff M Kvale P. Impact of comorbidity on lung cancer survival. Int J Cancer. (2003) 103:792–802. 10.1002/ijc.10882 12516101

[73] Dominguez-Ventura A Allen MS Cassivi SD Nichols FC Deschamps C Pairolero PC. Lung cancer in octogenarians: factors affecting morbidity and mortality after pulmonary resection. Ann Thorac Surg. (2006) 82:1175–9. 10.1016/j.athoracsur.2006.04.052 16996903

[74] Anker MS von Haehling S Landmesser U Coats AJS Anker SD. Cancer and heart failure-more than meets the eye: common risk factors and co-morbidities. Eur J Heart Fail. (2018) 20:1382–4. 10.1002/ejhf.1252 29943887

[75] Jain P Gutierrez Bugarin J Guha A Jain C Patil N Shen T. Cardiovascular adverse events are associated with usage of immune checkpoint inhibitors in real-world clinical data across the United States. ESMO Open. (2021) 6:100252. 10.1016/j.esmoop.2021.100252 34461483 PMC8403739

[76] Batra A Patel B Addison D Baldassarre LA Desai N Weintraub N. Cardiovascular safety profile of taxanes and vinca alkaloids: 30 years FDA registry experience. Open Heart. (2021) 8:e001849. 10.1136/openhrt-2021-001849 34952868 PMC8710909

[77] Ram P Tiu A Lo KB Parikh K Shah M. Trends in the prevalence of malignancy among patients admitted with acute heart failure and associated outcomes: a nationwide population-based study. Heart Fail Rev. (2019) 24:989–95. 10.1007/s10741-019-09808-y 31175492

[78] Plana JC Galderisi M Barac A Ewer MS Ky B Scherrer-Crosbie M. Expert consensus for multimodality imaging evaluation of adult patients during and after cancer therapy: a report from the American Society of Echocardiography and the European Association of Cardiovascular Imaging. Eur Heart J Cardiovasc Imaging. (2014) 15:1063–93. 10.1093/ehjci/jeu192 25239940 PMC4402366

[79] Hering D Faber L Horstkotte D. Echocardiographic features of radiation-associated valvular disease. Am J Cardiol. (2003) 92:226–30. 10.1016/S0002-9149(03)00546-0 12860232

[80] Iung B Delgado V Rosenhek R Price S Prendergast B Wendler O. Contemporary Presentation and Management of Valvular Heart Disease: The EURObservational Research Programme Valvular Heart Disease II Survey. Circulation. (2019) 140:1156–69. 10.1161/CIRCULATIONAHA.119.041080 31510787

[81] Chan J Rosenfeldt F Chaudhuri K Marasco S. Cardiac surgery in patients with a history of malignancy: increased complication rate but similar mortality. Heart Lung Circ. (2012) 21:255–9. 10.1016/j.hlc.2012.02.004 22386614

[82] Vahanian A Beyersdorf F Praz F Milojevic M Baldus S Bauersachs J. 2021 ESC/EACTS Guidelines for the management of valvular heart disease. Eur Heart J. (2022) 43:561–632. 10.1093/ejcts/ezac209 34453165

[83] Landes U Iakobishvili Z Vronsky D Zusman O Barsheshet A Jaffe R. Transcatheter Aortic Valve Replacement in Oncology Patients With Severe Aortic Stenosis. JACC Cardiovasc Interv. (2019) 12:78–86. 10.1016/j.jcin.2018.10.026 30621982

[84] Watanabe Y Kozuma K Hioki H Kawashima H Nara Y Kataoka A. Comparison of results of transcatheter aortic valve implantation in patients with versus without active cancer. Am J Cardiol. (2016) 118:572–7. 10.1016/j.amjcard.2016.05.052 27324159

[85] Berkovitch A Guetta V Barbash IM Fink N Regev E Maor E. Favorable Short-Term and Long-Term Outcomes Among Patients With Prior History of Malignancy Undergoing Transcatheter Aortic Valve Implantation. J Invasive Cardiol. (2018) 30:105–9. 29493511

[86] Mangner N Woitek FJ Haussig S Holzhey D Stachel G Schlotter F. Impact of active cancer disease on the outcome of patients undergoing transcatheter aortic valve replacement. J Interv Cardiol. (2018) 31:188–96. 10.1111/joic.12458 29166702

[87] Ming S Gang L Wei S. Thoracoscopic Lung Cancer Resection with Simultaneous Heart Valve Procedure. Heart Surg Forum. (2021) 24:E628–30. 10.1532/hsf.3937 34473025

[88] Hull MC Morris CG Pepine CJ Mendenhall NP. Valvular dysfunction and carotid, subclavian, and coronary artery disease in survivors of hodgkin lymphoma treated with radiation therapy. JAMA. (2003) 290:2831–7. 10.1001/jama.290.21.2831 14657067

[89] Malanca M Cimadevilla C Brochet E Iung B Vahanian A Messika-Zeitoun D. Radiotherapy-induced mitral stenosis: a three-dimensional perspective. J Am Soc Echocardiogr. (2010) 23:108.e1–2. 10.1016/j.echo.2009.08.006 19766451

[90] Glanzmann C Huguenin P Lütolf UM Maire R Jenni R Gumppenberg V. Cardiac lesions after mediastinal irradiation for Hodgkin's disease. Radiother Oncol. (1994) 30:43–54. 10.1016/0167-8140(94)90008-6 8153379

[91] Kim K Kim D Lee SE Cho IJ Shim CY Hong GR. Infective endocarditis in cancer patients - causative organisms, predisposing procedures, and prognosis differ from infective endocarditis in non-cancer patients. Circ J. (2019) 83:452–460. 10.1253/circj.CJ-18-0609 30555101

[92] Cosyns B Roosens B Lancellotti P Laroche C Dulgheru R Scheggi V. Cancer and infective endocarditis: characteristics and prognostic impact. Front Cardiovasc Med. (2021) 8:766996. 10.3389/fcvm.2021.766996 34859076 PMC8631931

[93] San Román JA López J Vilacosta I Luaces M Sarriá C Revilla A. Prognostic stratification of patients with left-sided endocarditis determined at admission. Am J Med. (2007) 120:369.e1–7. 10.1016/j.amjmed.2006.05.071 17398233

[94] Habib G Badano L Tribouilloy C Vilacosta I Zamorano JL Galderisi M. Recommendations for the practice of echocardiography in infective endocarditis. Eur J Echocardiogr. (2010) 11:202–19. 10.1093/ejechocard/jeq004 20223755

[95] Heit JA Spencer FA White RH. The epidemiology of venous thromboembolism. J Thromb Thrombolysis. (2016) 41:3–14. 10.1007/s11239-015-1311-6 26780736 PMC4715842

[96] Wendelboe AM Raskob GE. Global burden of thrombosis: epidemiologic aspects. Circ Res. (2016) 118:1340–7. 10.1161/CIRCRESAHA.115.306841 27126645

[97] Raskob GE Angchaisuksiri P Blanco AN Buller H Gallus A Hunt BJ. Thrombosis: a major contributor to global disease burden. Arterioscler Thromb Vasc Biol. (2014) 34:2363–71. 10.1161/ATVBAHA.114.304488 25304324

[98] Keller K Hobohm L Ebner M Kresoja KP Münzel T Konstantinides SV. Trends in thrombolytic treatment and outcomes of acute pulmonary embolism in Germany. Eur Heart J. (2020) 41:522–9. 10.1093/eurheartj/ehz236 31102407

[99] Barsoum MK Heit JA Ashrani AA Leibson CL Petterson TM Bailey KR. Is progestin an independent risk factor for incident venous thromboembolism? A population-based case-control study. Thromb Res. (2010) 126:373–8. 10.1016/j.thromres.2010.08.010 20833412 PMC2975753

[100] Shinagare AB Guo M Hatabu H Krajewski KM Andriole K Van den Abbeele AD. Incidence of pulmonary embolism in oncologic outpatients at a tertiary cancer center. Cancer. (2011) 117:3860–6. 10.1002/cncr.25941 21319153 PMC3131455

[101] Chuang YM Yu CJ. Clinical characteristics and outcomes of lung cancer with pulmonary embolism. Oncology. (2009) 77:100–6. 10.1159/000229503 19622900

[102] van Herk-Sukel MP Shantakumar S Penning-van Beest FJ Kamphuisen PW Majoor CJ Overbeek LI. Pulmonary embolism, myocardial infarction, and ischemic stroke in lung cancer patients: results from a longitudinal study. Lung. (2013) 191:501–9. 10.1007/s00408-013-9485-1 23807721

[103] Mulder FI Horváth-Puhó E van Es N van Laarhoven HWM Pedersen L Moik F. Venous thromboembolism in cancer patients: a population-based cohort study. Blood. (2021) 137:1959–69. 10.1182/blood.2020007338 33171494

[104] Cui YQ Tan XM Liu B Zheng Y Zhang LY Chen ZA. Analysis on risk factors of lung cancer complicated with pulmonary embolism. Clin Respir J. (2021) 15:65–73. 10.1111/crj.13270 32931143

[105] Ahlbrecht J Dickmann B Ay C Dunkler D Thaler J Schmidinger M. Tumor grade is associated with venous thromboembolism in patients with cancer: results from the Vienna Cancer and Thrombosis Study. J Clin Oncol. (2012) 30:3870–5. 10.1200/JCO.2011.40.1810 23008313

[106] Dentali F Ageno W Becattini C Galli L Gianni M Riva N. Prevalence and clinical history of incidental, asymptomatic pulmonary embolism: a meta-analysis. Thromb Res. (2010) 125:518–22. 10.1016/j.thromres.2010.03.016 20451960

[107] Li Y Shang Y Wang W Ning S Chen H. Lung cancer and pulmonary embolism: what is the relationship? A review. J Cancer. (2018) 9:3046–57. 10.7150/jca.26008 30210627 PMC6134828

[108] Giustozzi M Connors JM Ruperez Blanco AB Szmit S Falvo N Cohen AT. Clinical characteristics and outcomes of incidental venous thromboembolism in cancer patients: Insights from the Caravaggio study. J Thromb Haemost. (2021) 19:2751–9. 10.1111/jth.15461 34260816 PMC9290511

[109] Canonico ME Santoro C Avvedimento M Giugliano G Mandoli GE Prastaro M. Venous thromboembolism and cancer: a comprehensive review from pathophysiology to novel treatment. Biomolecules. (2022) 12:259. 10.3390/biom12020259 35204760 PMC8961522

[110] Nichols L Saunders R Knollmann FD. Causes of death of patients with lung cancer. Arch Pathol Lab Med. (2012) 136:1552–7. 10.5858/arpa.2011-0521-OA 23194048

[111] Ma L Wen Z. Risk factors and prognosis of pulmonary embolism in patients with lung cancer. Medicine. (2017) 96:e6638. 10.1097/MD.0000000000006638 28422863 PMC5406079

[112] Junjun L Pei W Ying Y Kui S. Prognosis and risk factors in older patients with lung cancer and pulmonary embolism: a propensity score matching analysis. Sci Rep. (2020) 10:1272. 10.1038/s41598-020-58345-4 31988400 PMC6985117

[113] Malgor RD Bilfinger TV Labropoulos N. A systematic review of pulmonary embolism in patients with lung cancer. Ann Thorac Surg. (2012) 94:311–6. 10.1016/j.athoracsur.2012.03.025 22626760

[114] Shinagare AB Okajima Y Oxnard GR Dipiro PJ Johnson BE Hatabu H. Unsuspected pulmonary embolism in lung cancer patients: comparison of clinical characteristics and outcome with suspected pulmonary embolism. Lung Cancer. (2012) 78:161–6. 10.1016/j.lungcan.2012.08.007 22959241 PMC3605722

[115] Sun JM Kim TS Lee J Park YH Ahn JS Kim H. Unsuspected pulmonary emboli in lung cancer patients: the impact on survival and the significance of anticoagulation therapy. Lung Cancer. (2010) 69:330–6. 10.1016/j.lungcan.2009.11.015 20007002

[116] Niksic M Redondo-Sanchez D Chang YL Rodriguez-Barranco M Exposito-Hernandez J Marcos-Gragera R. The role of multimorbidity in short-term mortality of lung cancer patients in Spain: a population-based cohort study. BMC Cancer. (2021) 21:1048. 10.1186/s12885-021-08801-9 34556072 PMC8461961

[117] Janssen-Heijnen ML Schipper RM Razenberg PP Crommelin MA Coebergh JW. Prevalence of co-morbidity in lung cancer patients and its relationship with treatment: a population-based study. Lung Cancer. (1998) 21:105–13. 10.1016/S0169-5002(98)00039-7 9829544

[118] Coebergh JW Janssen-Heijnen ML Post PN Razenberg PP. Serious co-morbidity among unselected cancer patients newly diagnosed in the southeastern part of The Netherlands in 1993-1996. J Clin Epidemiol. (1999) 52:1131–6. 10.1016/S0895-4356(99)00098-0 10580775

[119] Søgaard M Thomsen RW Bossen KS Sørensen HT Nørgaard M. The impact of comorbidity on cancer survival: a review. Clin Epidemiol. (2013) 5:3–29. 10.2147/CLEP.S47150 24227920 PMC3820483

[120] Islam KM Jiang X Anggondowati T Lin G Ganti AK. Comorbidity and survival in lung cancer patients. Cancer Epidemiol Biomarkers Prev. (2015) 24:1079–85. 10.1158/1055-9965.EPI-15-0036 26065838

[121] Navi BB Reiner AS Kamel H Iadecola C Okin PM Elkind MSV. Risk of arterial thromboembolism in patients with cancer. J Am Coll Cardiol. (2017) 70:926–38. 10.1016/j.jacc.2017.06.047 28818202 PMC5667567

[122] Giustozzi M Curcio A Weijs B Field TS Sudikas S Katholing A. Variation in the association between antineoplastic therapies and venous thromboembolism in patients with active cancer. Thromb Haemost. (2020) 120:847–56. 10.1055/s-0040-1709527 32369855

[123] Herrmann J. Vascular toxic effects of cancer therapies. Nat Rev Cardiol. (2020) 17:503–22. 10.1038/s41569-020-0347-2 32218531 PMC8782612

[124] Zaborowska-Szmit M Krzakowski M Kowalski DM Szmit S. Cardiovascular complications of systemic therapy in non-small-cell lung cancer. J Clin Med. (2020) 9:1268. 10.3390/jcm9051268 32349387 PMC7287714

[125] Mitchell JD Cehic DA Morgia M Bergom C Toohey J Guerrero PA. Cardiovascular manifestations from therapeutic radiation: a multidisciplinary expert consensus statement from the international cardio-oncology society. JACC CardioOncol. (2021) 3:360–80. 10.1016/j.jaccao.2021.06.003 34604797 PMC8463721

[126] Steingart RM Yadav N Manrique C Carver JR Liu J. Cancer survivorship: cardiotoxic therapy in the adult cancer patient; cardiac outcomes with recommendations for patient management. Semin Oncol. (2013) 40:690–708. 10.1053/j.seminoncol.2013.09.010 24331191

[127] Florido R Daya NR Ndumele CE Koton S Russell SD Prizment A. Cardiovascular disease risk among cancer survivors: the atherosclerosis risk in communities (ARIC) study. J Am Coll Cardiol. (2022) 80:22–32. 10.1016/j.jacc.2022.04.042 35772913 PMC9638987

[128] Al-Kindi SG Oliveira GH. Prevalence of preexisting cardiovascular disease in patients with different types of cancer: the unmet need for onco-cardiology. Mayo Clin Proc. (2016) 91:81–3. 10.1016/j.mayocp.2015.09.009 26602599

[129] Kocher F Fiegl M Mian M Hilbe W. Cardiovascular comorbidities and events in NSCLC: often underestimated but worth considering. Clin Lung Cancer. (2015) 16:305–12. 10.1016/j.cllc.2014.12.007 25659438

